# The Influence of Different Modes of Exercise on Healthy and Injured Tendons

**DOI:** 10.1155/2022/3945210

**Published:** 2022-09-09

**Authors:** Kaiyong Wang, Linlin Zhao

**Affiliations:** ^1^Guangdong University of Finance & Economics, Guangzhou, 510320 Guangdong, China; ^2^School of Kinesiology, Shanghai University of Sport, Shanghai 200438, China

## Abstract

Tendons are essential components of the musculoskeletal system that links the skeletal muscle to the skeleton. This dense connective tissue exhibits great plasticity. Therefore, research on the influence of types of exercise, including acute and long-term training, on the structural and mechanical properties of tendons in athletic and sedentary populations is of critical importance in the design of scientific-based exercise plans and effective tendinopathy treatment. Here, we review recent studies on the relationship between exercise and tendon health and tendinopathy repair to provide a general understanding of how exercise may reshape tendons.

## 1. Introduction

Tendon injury, especially patellar and Achilles tendon injuries, exhibits a high prevalence in professional athletes and impacts sports performance [1]. In sedentary populations and those who undergo repetitive overload of forearms during work, microruptures induced by acute mechanical stimulation and the subsequent unsolved chronic inflammation cause sporadic pain and disability [2]. Exercise has a close relationship with tendon homeostasis and injury repair. Proper exercise training may improve the mechanical function of tendons, while acute excessive loading poses a threat to tissue integrity. With increased understanding of tendon tissue characteristics, more effective exercise plan could be designed for different populations. Regarding the influence of exercise on tendons, it should be clearly noted that the modes, intensity, and frequency of exercise are all key parameters that need to be taken into account because different exercise executions involve various components of the musculoskeletal system and pose distinct challenges to energy metabolism and extracellular matrix (ECM) remodelling. On the other hand, the tendons in different locations of the body may experience force transmission to variable extents. Apparently, tendons that are mostly used and injured, for instance, the patellar tendon and Achilles tendon, attract most of the attention of researchers.

To better monitor and reflect the situation of tendons, many methodologies have been developed and utilized. The mechanical properties of the tendon including stiffness, tensile strength, cross-sectional area (CSA), slack length, and elasticity are assessed by protocols based on ultrasound [3]. In noninvasive ultrasound elastography, an ultrasound probe emitting external compression of the tissue is used to attain real-time measurement of the mechanical properties. Ultrasound has been applied in the in vivo assessment of different tendons [4, 5]. Moreover, measurement of the biomechanical properties of tendon grafts serves as a significant reference for surgical treatment with transplantation. Classical tensile testing machines and alternative video tracing measurements with more accuracy realized by digital cameras are applied in the examination of the mechanical properties of tendon grafts [6].

Different exercise styles often affect the rehabilitation care of patients with tendon injury. Previous studies have shown that patients with tendon injury usually progress from low-intensity exercise to high-intensity exercise, with increasing tendon load that patients must endure before fully resuming high-demand activities [7]. While tendon loading has been shown to improve patient symptoms, normalize tendon structure, and optimize functional performance, both acute and chronic Achilles tendon injuries have negative consequences when overloading or underloading [8]. Therefore, research on the influence of types of exercise, including acute and long-term training, on the structural and mechanical properties of tendons in athletic and sedentary populations is of critical importance in the design of scientific-based exercise plans and effective tendinopathy treatment. Here, we review recent studies on the relationship between exercise and tendon health and tendinopathy repair to provide a general understanding of how exercise may reshape tendons.

## 2. The Physiology and Pathology of Tendon

### 2.1. The Anatomical Structure of the Tendon

**Figure 1:**
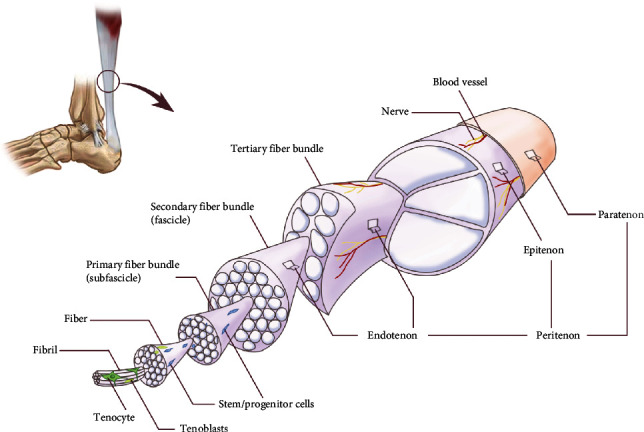
The anatomy structure of tendon. The cell composition including tenocytes, tenoblasts, and tendon-derived stem cells (TDSCs) and the fibre organization of tendon and the enveloping structure are presented.

Before investigating the relationship between exercise and tendon tissue, a comprehensive understanding of the physiology and pathology of tendons is necessary (Figure 1). Located in many parts of the body, tendons are a crucial component in the locomotion system, which connects skeletal muscle and bone to play a mechanical stimulation transition role. In addition to force transmission, the tendon also absorbs shock and stores energy. Frequent use and heavy loading reduce the energy the tendon absorbs but increase the adaptivity to heavy loading, whereas a low rate of loading increases the viscosity of the tendon and the energy it absorbs. Given its abundance in ECM, normal tendon function largely depends on the homeostatic metabolism of these extracellular constituents. In addition to the ECM, the process of tendon development and regeneration is tightly regulated by transcription factors and growth factors belonging to different signalling pathways [9]. In the three germ layers during embryonic development, the origination of tendon includes ectoderm and mesoderm. Craniofacial tendons develop from neural crest cells of the ectoderm [10]. While tendons in the limb bud derived from the tendon progenitor cells in mesoderm, whose development are regulated by the signals of ectoderm and bone morphogenetic protein (BMP) signaling [11].

Linking the skeletal muscle to the bone, the tendon transmits the mechanical force from the skeletal muscle to the bone, enabling locomotion. Similar to the structure of the skeletal muscle, the fibrils in tendons have a hierarchical relationship. The fibrils composed of type I collagen in a triple-helical form fibre, then fascicles, and finally the tendon [12]. To form fibrils, tropocollagen is synthesized in fibroblasts secreted and cleaved extracellularly to become collagen. As the highest hierarchy in the tissue, fascicles are enfolded by a mesh of loose connective tissue, namely, endotenon. The endotenon structure enables the compartmentalization of fibre so that other components including blood vessels, nerves, and lymphatics can infiltrate and nurture the fibre. The epitenon, surrounding the whole tendon, is continuous throughout the inner surface with the endotenon. In the outermost layer, fatty, the areolar tissue is penetrated by nerves and vessels and surrounded by paratenon, which allows the tendon to move freely in the surrounding tissues without friction. The thickness and organization of the fibril as well as the number, size, and orientation of the collagen fibre coordinately determine the strength and viscoelasticity of the tendon. In addition to type I collagen, the other ECM components in the fascicles include proteoglycans, glycoproteins, elastin, and many other types of collagens, which undergo reprogramming and remodelling in the context of long-term mechanical stimulation or injury healing [13]. As the cell type that dominates in mature tendon tissues, tenocytes take a great responsibility in ECM production, maintenance, and remodelling. It is through regulating the extracellular environment that tenocytes sense and respond to mechanical stimulation [14]. During degradation, the enzymes for protein cleavage secreted out of the cells so that the resulted digestion products can be phagocytosed by tenocytes. These recycled collagen fibrils may be used for new ECM formation intracellularly [15].

Away from the middle part of the tendon, the heterogeneity increases given the formation of junctions that link the tendon to the skeletal muscle and the skeleton, namely, the myotendinous junction and osteotendinous junction, respectively. Between the muscle and the tendon, the myotendinous junction receives stress during contractile force transmission, where the fibre from the tendon inserts into the muscle body to increase the stability of the structure. The fibrocartilaginous tissue that connects the tendon and the skeleton is also called an enthesis. A large amount of type II collagen produced by chondrocyte-like cells in this area shapes the different mechanical properties and pathogenesis of enthesis and tendon, although injuries occurring in both areas are not easily repaired completely.

### 2.2. Blood Vessels and Nerves in the Tendon

Although the situations are not all the same in all the tendons in the body, generally, the tendons are relatively poorly vascularized with more dependency on synovial fluid diffusion to provide nutrition. The myotendinous junction, enthesis, and surrounding connective tissue such as the paratenon serve as the origin of blood supply for the tendon [16]. Blood vessels from the perimysium, periosteum, paratenon, and mesotendon penetrated the endotenon and epitenon into the fibre and those blood vessels from the paratenon predominating in the middle part of the tendon. As a crucial source of nutrition and an approach to metabolite exchange, the blood vessel pattern changes when tendon injury results from acute friction, rupture, torsion, or compression, especially in frequently used tendons such as Achilles tendons. The phenotypes are controversial. Specifically, hypovascularity was found in some degenerated or ruptured tendons, whereas large blood vessels were found in some tendinopathy cases. Of note, blood vessels are not always a good sign for tendon injury healing. The paratenon is also an origin of sensory nerves for the tendons. The nerve plexuses penetrate the epitenon and branch inside the tendon fibre [17]. The innervation of tendon tissue proper is described as relatively scarce [18].

### 2.3. Cell Composition of Tendon

Mature tendons are composed of dense connective tissue that is hypocellular, with most of the cells in the tissue being active and being highly proliferative and fibroblast-like tenoblasts and terminally differentiated tenocytes. The tenocytes are spindle-shaped flat cells lying in rows between collagen fibres, forming an exquisite three-dimensional network in the ECM to maintain cell–cell communication [19]. The highly developed rough endoplasmic reticulum in the tenoblasts allows the efficient production of collagen and other ECM. Tenocytes are tenospecific fibroblasts responsible for the production, maintenance, repair, and modification of the ECM [20]. Scleraxis (Scx) and tenomodulin (TNMD), as relatively specific molecular markers of tendons, are often used for the identification of tendon cells, and these proteins play a central role in the development and maturation of tendons [21]. Cserjesi et al. discovered Scx in 1995 using the yeast two-hybrid system for cell-type-specific proteins. Scx is expressed in early embryonic development and regulates tendon maturation [20]. Tenomodulin is a class of type II transmembrane proteins first reported by Shukunami et al. that can regulate the proliferation of tendinocytes and the maturation of collagen fibre [20, 22]. With the explosive-increase in the stem cell research field and proposed bioengineering tissue repair concepts, the search for a cell population with excellent regenerative capability in tendon tissue is ongoing. The identification of tendon-derived stem cells (TDSCs) in animal models and humans is necessary for the elucidation of the cell hierarchy development trajectory of tendons and the identification of new cell sources for bioengineering approaches in tendon injury treatment.

TDSCs were first isolated from human hamstring and mouse patellar tendons in 2007, after which TDSCs were identified in many other animals including rats and rabbits. TDSCs express various markers, including octamer-binding transcription factor-4 (Oct-4), Nanog, nucleostemin (NS), stage-specific embryonic antigen (SSEA)-4, c-Myc, and SRY-box transcription factor (Sox) 2 [23]. Here, we focus on the relationship between exercise interference and tendons, and more information about the labelling of the TDSCs can be found elsewhere.

### 2.4. Tendon Mechanobiology

Macroscopically, exercise stimulates the tendon mechanistically. Upon activation of molecular signalling pathways, mechanical stimulation signals are conveyed by the ECM deep into the cell and converted to intracellular biological signals by mechanosensitive receptors in the cell plasma membrane. Investigations of the response of tendon cells to mechanical stimulation and the underlying mechanism are of significance for guiding exercise interference in tendon remodelling and tendinopathy treatment. Conversely, the observation and summary of the adaptation of tendons to mechanical loading would enlighten research on the pathogenesis of tendinopathy. With the development of different modes of mechanical stimulation including compression and tension and bioengineering methods to construct cultured tendon tissue, an increasing number of molecules and related signalling pathways involved in the mechanical regulation of the tendon have been reported.

Composed of tendon cells, strong collagen fibre, and diverse noncollagen ECM, tendons are dynamic responders to frequent mechanical loads. ECM remodelling during this process influences cell function. The secretion and degradation of the ECM in tendons change sensitively after mechanical stimulation to adapt to the environment and maintain homeostasis. Appropriate mechanical force from the structures around the tendon is necessary for the development and maturation of the tendon. Among the molecules that participate in the regulation of tendon dynamics, scleraxis (Scx) has been studied most. This transcription factor is expressed specifically in progenitors and cells in all tendons. Scx deficiency in mice results in limited use of all paws and back muscles and a complete inability to move the tail [24]. Fibroblast growth factor (FGF) in the MAPK/ERK signalling pathway and transforming growth factor-beta (TGF*β*) in the SMAD2/3 signalling pathway are reported to regulate the expression of Scx [25, 26]. During injury repair, numerous growth factors and cytokines are expressed and secreted to fulfil the proliferation and remodelling of tendons. Crucial transcription factors including Scx and Mohawk (Mkx) are upregulated after injury [27]. The upregulation of the transcription factors paves the way for alteration of the extracellular environment. Early growth response protein 1 (EGR1) is required for the transcription of Col1a1 [28]. Fibroblast growth factors (FGFs) also promote the production of collagens [29]. TGF*β* signalling functions in ECM organization induced tenocyte morphogenesis and the formation of myotendinous junctions [19, 30]. In addition to molecules that are directly related to tendon cells, other cell types such as immune cells and endothelial cells and their corresponding factors, including vascular endothelial growth factor (VEGF) and platelet-derived growth factor (PDGF), are critical to confer recovery ability to the tendon [31, 32]. Further mechanistic research would reveal more unknown factors with the potential to be treatment targets to develop better strategies for tendon injury repair.

## 3. Exercise and Tendons

### 3.1. Acute Exercise and Tendons

The immediate effect of a certain mode of exercise on tendons is worthy of exploration for several reasons. Acute exercise with excessive dose and intensity may expose the tendon on the risk of strain injury, partial to complete rupture, and structural destruction of the myotendinous junction [33, 34]. Measurements and analysis of the morphological and mechanical property changes in tendon tissue after acute exercise serve as the basis to understand the mechanism of acute tendon injury and thus to develop effective treatment intervention for exercise and nonexercise populations. It is also important to note that the same exercise mode and dose may have different effects on tendons in healthy and diseased situations due to their tolerance and the capacity to restore after mechanical loading. Furthermore, the accumulation of knowledge of tendon mechanical properties is valuable for the prevention of acute tendon injury and consequential disability and pain.

The stiffness of the Achilles tendon is reduced after maximal voluntary contractions [35, 36]. However, during exercise in the stretch shortening cycle mode, including running and hopping, the stiffness of the Achilles tendon did not significantly change [37]. After acute eccentric exercise, the diameter of the Achilles tendon is reduced significantly [38, 39]. The Achilles tendons of 14 participants who underwent acute eccentric heel drop exercise exhibit significantly increased free tendon length and strain [40]. Generally, the Achilles tendon responds to prolonged stretching and repeated maximal isometric contractions of the triceps surae with transient reduced stiffness and hysteresis, whereas the morphological and mechanical properties do not seem to change after stretch-shortening cycle exercise, such as treadmill running and hopping [41]. It is concerning that the reduced stiffness of the tendon resulting from acute overload may increase the probability of tendon injury.

In addition to morphological analysis, for in-depth knowledge of the alterations in tendons after acute exercise, protein synthesis was examined in tendons. To assess protein synthesis in human subjects, isotope labelling through intravenous injection was employed, and the labelling of target proteins was quantified by gas chromatography–mass spectrometry for quantification. The study showed a rapid increase in collagen synthesis in the patellar tendon after strenuous exercise in the young male volunteers, and the levels peaked at 24 hours post exercise [42]. Another assessment of the expression of extracellular matrix components and related factors revealed no change in the mRNA levels of collagens and noncollagenous matrix proteins, whereas mRNA expression levels of insulin-like growth factor IEa (IGF-IEa) decreased. The biopsies in this research were collected from 31 healthy young men who performed one-leg kicking at 67% of the maximum workload for 1 hour [43]. Given that an increase in collagen protein was detected in other studies, the lack of a change in mRNA expression may imply that acute exercise with a certain intensity may regulate the cleavage of procollagen and metabolism of extracellular collagen. It would be intriguing to explore the alteration in intracellular and extracellular collagen metabolism after acute exercise.

In an observation of calf Achilles tendon, isolated eccentric and concentric loading with the addition of 20% body weight of the Achilles tendon was exerted by the two limbs separately. Sonograms collected 3-24 hours after exercise showed that both loading conditions resulted in decreased normalized Achilles tendon thickness, whereas eccentric loading induced a significantly greater Achilles tendon thickness than concentric loading. However, the transient change after the two modes of exercise completely recovered during a similar time course [38]. An experiment involving 6 males and 4 females reported that there is no difference in the stiffness of Achilles tendon after a single round of hopping exercise with the assessment technique ultrasonography and MRI, although the maximum tendon force during maximum voluntary contraction was reduced after the exercise [44]. Although the number of subjects in this report was relatively small, the findings suggested that in healthy individuals and potentially in all cases, a single round of physiological stress level exercise did not result in mechanical fatigue of the Achilles tendon.

Stretching is also necessary to adjust the state of skeletal muscle and tendons before and after exercise. Dissection of the function of stretching in tendon movement is important to obtain a proper warm-up effect before and after relaxation of concentrated mechanical loading. A study with 14 participants (7 men and 7 women) showed that the range of motion and maximum voluntary contraction increased after 5 rounds of 60 seconds of static stretching, whereas the muscle-tendon stiffness decreased immediately but not at 5 or 10 minutes poststretching. The mechanical changes in the muscle-tendon junction were attributed to changes in muscle elongation instead of changes in tendon morphology [45].

Although experimental and clinical data have continued to accumulate since the scientific management of the musculoskeletal system attracted the attention of the public, studies on the relationship between acute exercise and tendon health remain blurry and controversial given variations in the methodologies and exercise modes and doses. Moreover, for exercise and nonexercise populations or young and old populations, the stimulation conditions established during the test should differ, as the tendons of these populations are manipulated in distinct manners via different mechanical properties. Regardless of the measurement used for the tendon itself, real-time, high-resolution observation and reflection of the mechanical state of the myotendinous and osteotendinous junction remain lacking. Acute stretching and shortening of the tendon also place great stress on the junction part of the tendon, and the environment is harsh for repair if rupture occurs due to the diversity and low proliferation activity of the cells. It is important to develop suggestions for injury prevention based on a basic understanding of the mechanical characteristics; moreover, knowledge on the junction is also critical.

### 3.2. Long-Term Exercise and Tendons

Compared with a single bout of exercise, long-term training with a certain frequency is more likely to reshape locomotion system components. In a study involving 40 Chinese subjects 19 to 25 years old who were divided into two groups subject to frequent or infrequent exercise, the mean thickness of the Achilles tendon of subjects undergoing frequent-exercise was significantly greater than that of subjects undergoing infrequent- exercise. The same result of the CSA of the tendon was reported in the dominant ankle [46]. Similarly, increased CSA and stiffness of the tendon were reported after prolonged eccentric training [47, 48]. Collectively, the increased CSA and stiffness of the tendon was regarded as a positive sign because this would allow the tendon to sustain greater mechanical loading and store elastic energy more efficiently. In long term, the morphological and mechanical changes of tendons made by long-term training reduce the stress that the tendon absorbs and decrease the probability of tendon injury when overload occurs. Research comparing of male athletes and Achilles tendon rupture patients through magnetic resonance imaging and maximal isometric plantar flexion force measurements demonstrated that the CSA of tendons normalized by body weight was greater in athletes accepting intermittent high loads, and no obvious structural or loading property differences were noted between the Achilles tendons of rupture patients and the athlete group. The Achilles tendon of rupture patients did not undergo greater force or stress during maximal voluntary isometric plantar flexion than that of the athletes [49]. To investigate the effect of habitual long-term training on human tendons, the researchers assessed the tendon elongation and CSA of the patellar tendon and Achilles tendon of female runners and nonrunners, (*n* = 10). The patellar tendon and Achilles tendon CSA are all comparable in trained and untrained women [50].

The effects of long-term training in a healthy athlete population on tendons may also result in differences in exercise performance. A study of 26 healthy recreational long-distance runners assessed alterations in tendon force and stiffness resulting from long-term resistance training and running economy [51]. The exercise group was subject to an additional a resistance training intervention in their previous running training for 14 weeks, whereas the training of the control group remained the same. Ultrasonography results indicated that the maximum plantar flexion muscle and tendon-aponeurosis stiffness were significantly increased in the exercise group. In the exercise group, better running economy was reflected by reduced oxygen consumption and energy cost.

Along with other components in the musculoskeletal system, the tendon is a highly adaptive tissue with dynamic changes occurring inside after the stimulation of mechanical loading. A meta-analysis focusing on the adaptation of human tendons to mechanical loading reviewed different studies on human tendon adaptation to mechanical loading [52]. Conclusions from such review may serve as an important reference given that these findings provided more statistical evidence for one topic. They found in the reviewed research that the exercise intervention-induced changes in tendon stiffness seem to be more attributed to material instead of morphological properties. In this analysis, the authors demonstrated that loading magnitude is the key element in the loading regimens in contrast to muscle contraction type. The results from this analysis advocate for a high loading intensity and an intervention duration that is longer than 12 weeks for an effective exercise intervention when studying the influence of exercise on tendon properties.

Diverse animal models simulating human exercise were devised to investigate how exercise influences healthy or injured tendons. The horse is a large animal model used to study human tendon physiology and pathology. Research observing two groups of female horses with high or low intensity exercise training for 18 months found that the collagen fibril diameter of the superficial digital flexor tendon decreased in the high intensity group, but no change in collagen content was observed. Compared with the injury-prone equine superficial digital flexor tendon, the rarely injured common digital extensor tendon showed lower water content and higher elastic modulus after long-term, high-intensity training; however, no signs of degeneration or mechanical property changes were noted in the superficial digital flexor tendon [53]. In one study, 24-month-old rats were divided into three groups based on a treadmill exercise protocol of sedentary, moderate and high intensity for up to 12 months. However, the exercise level did not have a significant effect on the elastic modulus parameter of the tail tendon; however, a decreasing trend was noted at moderate and high intensity compared with the sedentary control group [54]. This result suggested that even long-term exercise was unable to induce a systemic effect on the mechanical properties of old tendons, the structures of which become disorganized during the process of ageing. It is not practical to expect old tendons to exhibit properties of young tendons when exercise is employed as the only intervention after the structure of the tendon has been completely transformed.

Given its regulatory function in fibroblast proliferation and type I collagen synthesis, the TGF-*β* superfamily is believed to be an essential signalling pathway that participates in the adaptation of tendons to exercise stimulation [55]. Limb formation failure in TGF-*β* receptor inactivation mice provides strong evidence. Nevertheless, the spatiotemporal control of gene activation and inactivation by genetically modified mouse models is urgently needed to investigate the function of TGF-*β* function in adult tendons during types of loading interventions.

A common understanding of the mechanism in tendon physiology and pathology depends on the uniformity of the criteria employed in the research methodology. A myriad factors of factors should be taken into account when reviewing different research results given that the assessment approach and resolution, the exercise dose and intensity, the race, age, and gender of the subjects and many other factors may lead to variable conclusions. It was even suggested by some scientists that conditioning exercises should be performed to standardize the load history of tendons before in vivo sonographic measurements of tendon thickness. According to their findings from 30 healthy male participants, conditioning of the Achilles tendon via resistive ankle exercise induced alterations in tendon structure that improved correlations between tendon thickness and body anthropometry [56]. This detailed exploration of the experimental design provided information for a more scientific protocol to examine the relationship between tendon physiology and exercise.

In the observations of tendon adaptation to acute or habitual mechanical loading, the difference between males and females cannot be neglected. Of note, women are more susceptible to soft tissue injury, but the reason is unknown. It was reported that after a bout of acute exercise, collagen synthesis in tendons of men was upregulated but the increase in tendons of women was less profound or absent. Additionally, the patellar tendon of men increased in size after long-term training, whereas no alterations were noted in women. A lower mechanical strength and reduced rate of connective tissue formation in tendons of women were proposed to explain the increased risk noted in women [57]. Regarding the biological mechanism, ethinyl oestradiol, a synthetic oestrogen, inhibits the acute exercise-induced collagen synthesis in female tendons [58]. More specific mechanisms should be studied to resolve the increased risk of tendon injury in women (Table 1).

## 4. Exercise and Tendon Injury

Tendon pathologies can be divided into chronic injury and acute injury with partial or complete tendon rupture in different specific locations of the tendon. Tendon repair injury includes three overlapping stages of inflammation, proliferation and remodelling [9]. During an inflammatory phase lasting several days, neutrophils recruit to the site of injury and macrophages clear necrotic debris [59]. About two days after the injury, tendinocytes are recruited to the damaged area and stimulate proliferation. Meanwhile, macrophages transform from phagocytosis to repair and promote fibroblast proliferation and guide ECM remodelling by releasing chemokines and growth factors [60]. One to two months after the injury, the synthesis of type I collagen begins to dominate, and the tendon injury enters the remodelling stage, which lasts for more than a year, and the repaired tissue becomes scarring [61]. Sudden rupture of tendons resulting from inappropriate locomotion system utilization is followed by a cascade of repair featur including inflammation, tendon tissue cell proliferation, and ECM remodelling. Characterized by long-term pain and impaired mobility, chronic tendon injury, which is also known as tendinopathy, is often recognized as resulting from tissue overuse. Chronic inflammation and ECM reprogramming during tendinopathy are closely related to the pathogenesis, but the exact mechanism remains to be explored. Furthermore, the mechanisms underlying the healing and the available interference for tendon degeneration also depend on the elucidation of the pathogenesis of tendon injury.

All the tendons in the body can be injured, among which the tendons that are used more often and perform more mechanical loading are injured more frequently. Triceps tendinopathy occurs frequently in throwing athletes and results from the insertion of the tendon into the olecranon. Patellar injuries, as well as Achilles injuries, are common in athletes involved in repetitive jumping, kicking, and running. Wrist extensor tendinopathy is associated with eccentric loading of the forearm muscle in throwing movements. Overload and repetitive improper employment of the musculoskeletal system and the sequential unsolved chronic inflammation, which impedes the proliferation of cells and remodelling of the tissue, together make the recovery of tendinopathy complicated [62].

Factors, including acute tearing, oxidative damage, and accumulation of microtears, would cause structural destruction of the tendon matrix. The matrix synthesized after injury acts as a template for sequential matrix remodelling. As a result, fibrotic scars with different cell and ECM compositions compared with normal tendon tissue appear. The poor mechanical properties of the scar affect the elongation and energy storage of the tendon [63].

## 5. Exercise in the Treatment of Tendon Injury and Degeneration

**Figure 2:**
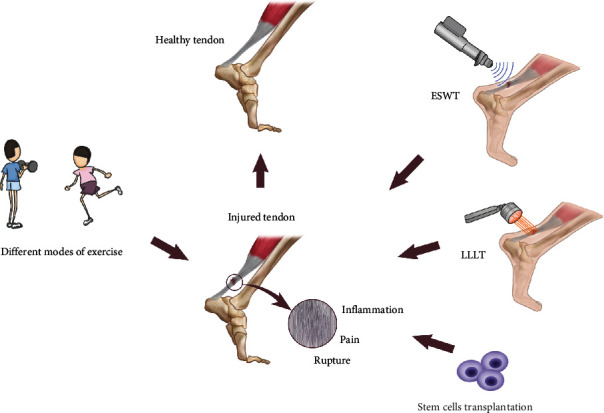
Exercise combined with other approaches in tendon treatment. Different modes of exercise including eccentric exercise and techniques such as extracorporeal shock wave therapy (ESWT), low-level laser therapy (LLLT), and tendon-derived stem cells (TDSCs) transplantation are being applied in the treatment of tendinopathy.

The current treatments for tendon injury mainly include conservative therapy and surgery followed by allogenic transplantation. In the conservative strategies, eccentric exercise therapy and other interventions, including extracorporeal shock wave, ultrasound, and low-intensity lase treatment, are utilized [23] (Figure 2).

A scientifically developed exercise protocol was demonstrated to be effective in Achilles tendon rehabilitation by incrementally increasing the rate and magnitude of tendon loading. To propose guidelines on how to improve the function of tendons, 8 healthy young adults performed a series of rehabilitation exercise according to the plan. They researchers found that Achilles tendon loading increased when a set of isolated ankle movements or multijoint movements was performed [8]. The finding demonstrates that tendon rehabilitation could be achieved through appropriate exercise given the striking plasticity of tendon tissue. This information has encouraged clinicians and researchers to optimize the exercise training protocol in tendon treatment. Given its potential benefits on the rehabilitation of Achilles tendinopathy, biomechanical research of eccentric exercise on tendon physiology and pathology should be performed to develop an optimal treatment protocol. Real-time recordings of 16 healthy subjects performing one-legged full weight bearing ankle plantar and dorsiflexion exercises revealed no difference in Achilles tendon loads; however, the surface electromyography of the lower leg muscles was reduced [64]. The researchers argued that although the tendon loads are similar, the tendon is vibrated at higher frequencies during the eccentric phase than during the concentric phase. This phenomenon may at least partially explain the effect of eccentric exercise in tendinopathy treatment. In addition to the widely used eccentric exercise, the treatment effect of other types of exercise was also tested. In a clinical study comparing progressive tendon-loading exercise (PTLE) with eccentric exercise therapy (EEP), 76 patellar tendinopathy patients were divided into two groups that received two modes of training [65]. The researchers reported that PTLE resulted in a significantly better clinical outcome after 24 weeks than EET. Such exploration broadens clinicians' horizons and provides superior choice than the conventional eccentric exercise currently recommended.

Extracorporeal shock wave therapy (ESWT) is a physiotherapeutic intervention utilized in tendinopathy treatment. The superiority of the effectiveness of ESWT compared with conservative treatment, such as inflammatory drugs, exercise programs alone, or the use of knee traps in patellar tendinopathy treatment, has been demonstrated [66]. Recently, the influence of ESWT combined with exercise on tendinopathy treatment has been investigated. Thirty-four male athletes suffering from patellar tendinopathy for more than 3 months were divided into an exercise control group who received long-term eccentric exercise (i.e., 12-week single legged decline squat exercise) and a combined group who received a weekly session of ESWT in the initial 6 weeks along with the same exercise plan as the control group. Although a significant reduction in tendon stiffness, an increase in tendon strain and a reduction of in pain intensity were found after eccentric exercise, the addition of ESWT did not seem to have an obvious effect on the clinical outcome [67]. A similar result was found regarding the effect of ESWT on rotator cuff tear repair. Thirty-five patients underwent ESWT for 6 weeks after surgery, whereas the control group did not. The examination included computed tomographic arthrography 6 months after surgery and a minimum one-year follow-up. No significant difference was noted between the two groups [68]. The mechanism was considered to involve the cellular changes induced by mechanotransduction triggered by ESWT [69]. On the other hand, an in vitro positive effect of extracorporeal shock waves on the cell behaviour of tendon cells was demonstrated. In cultured primary human tenocytes, shock wave treatment promoted cell proliferation and collagen synthesis [70]. The differentiation of human tendon-derived stem/progenitor cells was also accelerated by ESWT [71]. Based on in vivo and in vitro results of ESWT research, a deeper understanding of the mechanism of how ESWT influences tendon tissue is expected to facilitate improved clinical guidelines.

Low-level laser therapy (LLLT) has also attracted the attention of scientists in tendon injury repair. In a rat Achilles tendon injury model achieved by surgical hemi-transection of the Achilles tendon, different dosages of laser treatment were combined with running exercise for 3 weeks for injury repair. The results showed that rats receiving laser irradiation had less load-relaxation than control rats [72]. During the compensatory overload of the plantar muscle in rats, infrared laser irradiation improved collagen organization in tendons [73]. Combined with adipose-derived mesenchymal stem cell transplantation in rat calcaneal tendon injury, LLLT also hastened collagen organization during tendon repair [74]. Moreover, LLLT functions in different phases in tendon repair including promoting angiogenesis during the inflammatory phase and reducing the inflammatory response during the remodelling phase [75].

In recent decades, burgeoning evidence in animal models and human clinical trials has demonstrated that stem cells derived from different tissues can be utilized in tissue injury repair. The hypocellularity and hypovascularity in the mature tendons makes their self-repair capability very limited. The ECM-enriched tissue environment gives rise to fibrosis and scar tissue with inferior mechanical properties. All these characteristics make the identification and application of TDSCs in the repair of tendon injury urgent and attractive.

Multipotent mesenchymal stem cells (MSCs) are commonly used in tissue regeneration given the abundance of cell resources. The application of MSCs in tendon repair has been tested, and the cells serve to increase early tendon strength and decrease overall healing time [76]. TDSCs are thought to be more suitable for the regeneration of tendons given their high proliferative activity and more mature tendon-like differentiated results. TDSCs promote tendon repair in a rat patellar tendon window defect model and facilitate improved cell alignment and collagen organization [77]. With the assistance of tissue engineering, accurate cell delivery and prolonged retention have been gradually achieved [78]. However, the acquisition of sufficient TDSCs with stable potent properties will still depend on further developed cell culture and augmentation techniques. Although the therapeutic use of TDSCs in tendon treatment is promising due to the potent differentiation capability and plasticity of stem cells, methods require further development given that it is difficult to maintain stem cell properties during the long culture and modification process. Development of an ideal marker combination, standardization of cell culture, amplification and manipulation methods, and the development of transplantation strategies are all noteworthy points.

## 6. Conclusions

Collectively, we discuss the physiology of tendons and the pathology of tendinopathy. Although the basic anatomical structure of the tendon is well understood, a significant amount of mechanistic information remains unknown. With the exception of tendon cells and the ECM, the composition and dynamics of other cell types, including endothelial cells and pericytes of blood vessels, neurons, and immune cells, in healthy or injured tendon tissue must be further elucidated by newly developed methods, such as intravital confocal microscopy. Acute exercise may lead to transient alterations in collagen synthesis and the mechanical properties of tendons. Acute overloading increases the risk of tendon rupture, whereas proper static stretching prepares the tendon for better mechanical loading performance. The reshaping of tendons based on long-term frequent exercise increases their adaptability to mechanical load stimulation, reduces the probability for injury, and improves better locomotion economy. In investigations on effects of exercise on tendons, the modes and intensity of exercise and the gender of the subjects are all should not be ignored regardless of whether the subjects are athletic or sedentary. Distinct exercise interventions based on the characteristics of the population are recommended for tendon rupture prevention and tendinopathy treatment. Exercise, especially eccentric exercise, is regarded as effective in restoring tendon function. More exercise mode options with logical design are expected. Exercise rehabilitation combined with various technologies, including ESWT, LLLT, tissue engineering, and TDSTs, represents a powerful independent approach or follow-up treatment after surgery.

## Figures and Tables

**Table 1:** The influence of different modes of exercise on tendons.

Classification of exercise	Type of exercise	The influence on the tendon
Acute exercise	Running and hopping	The stiffness of the Achilles tendon did not significantly change
	Acute eccentric exercise	The diameter of the Achilles tendon is reduced significantly
	Acute eccentric heel drop exercise	Free tendon length and strain increased
	One-legged kicking exercise	An increase in collagen protein was detected, no change in the mRNA levels of collagens
	A single bout of hopping exercise	No difference in the stiffness of Achilles tendon

Long-term exercise	Prolonged eccentric training	CSA and stiffness of the tendon increased
	An additional a resistance training intervention after running training (14 weeks)	The maximum plantar flexion muscle and tendon-aponeurosis stiffness were significantly increased
	High intensity exercise (18 months, female horses)	The collagen fibril diameter of the superficial digital flexor tendon decreased
	Moderate and high intensity treadmill exercise (12 months, 24-month-old rats)	Long-term exercise was unable to induce a systemic effect on the mechanical properties of old tendons, the structures of which become disorganized during the process of aging
